# Clinical case of Cor triatriatum sinister, a dilemma of anticoagulation: A case report and literature review

**DOI:** 10.1002/ccr3.8908

**Published:** 2024-06-25

**Authors:** Alireza Arzhangzade, Mahmood Zamirian, Salma Nozhat, Sasan Shafei, Roozbeh Narimani Javid, Sarvenaz Salahi, Soorena Khorshidi

**Affiliations:** ^1^ Department of Cardiology, School of Medicine Shiraz University of Medical Sciences Shiraz Iran; ^2^ Skull Base Research Center, Loghman Hakim Hospital Shahid Beheshti University of Medical Sciences Tehran Iran; ^3^ Student Research Committee Hamadan University of Medical Sciences Hamadan Iran; ^4^ Minimally Invasive Surgery Research Center Iran University of Medical Science Tehran Iran

**Keywords:** anticoagulant, atrial fibrillation, CHA2DS2‐VASc, Cor triatriatum sinister

## Abstract

Cor triatriatum is a rare congenital heart abnormality in which a membrane separates the left atrium (LA; sinister) or the right atrium (dexter) into two compartments. It is also a long‐forgotten cause of atrial fibrillation (AF) and substantially higher rates of blood stagnation, particularly proximal to the additional septum in the LA. In this case report, we faced a CHA2DS2‐VASc score of 1 in patients with non‐valvular AF due to Cor triatriatum sinister (CTS). The decision to start anticoagulants in this particular case was controversial, so we reviewed the literature to assess and address it. We present our case and discuss the indication of anticoagulants in this unique clinical scenario, accompanied by a literature review. Facing this dilemma of starting anticoagulants in special cases of CTS and AF should be individualized and need more investigation. However, till this moment, based on similar reports, it seems to be rational to consider CTS Per se as an additional risk stratification marker beyond the CHA2DS2‐VASc score start anticoagulant until the surgical resection. Considering CTS as the sole indication of anticoagulant in patients with normal sinus rhythm is a complex matter that needs further investigation.

## 1 INTRODUCTION

Cor triatriatum is an uncommon congenital cardiac anomaly characterized by the presence of a membrane that divides the left atrium (LA; sinister) or right atrium (dexter) into two distinct compartments. It accounts for around 0.4% of congenital heart disease (CHD).^1^, ^2^

Typically, the chamber located dorsally and obliquely inferior to the chamber of the left ventral atrium is the site where the pulmonary veins drain. The two sections of the LA are connected through openings in the membrane that vary in size and number.^3^

The surgical intervention for Cor triatriatum involves the removal of the intra‐atrial membrane. A few surgical facilities have documented commendable long‐term survival rates following membrane resection, accompanied by a low risk of postoperative reintervention.^4^, ^5^ This procedure may even be contemplated for patients who do not exhibit any symptoms but have a significant gradient across the membrane, typically falling within the 8–10 mmHg range. In this study, we introduce a case of a young adult female who has been diagnosed with persistent paroxysmal palpitation and newly diagnosed AF and Cor triatriatum.^6^

In this case report, we encountered a CHA2DS2‐VASc score of 1 in a patient with non‐valvular atrial fibrillation (AF) due to Cor triatriatum sinister (CTS). The decision to start anticoagulants in this particular case was controversial, so we reviewed the literature and assessed and addressed the most appropriate treatment strategy in similar cases.

### 1.1 Case history

The patient was a 26‐year‐old woman in good health with no previous medical history, complaining of multiple episodes of paroxysmal palpitation over the preceding year. There were no documented comorbidities in her medical history, including heart failure, hypertension, dyslipidemia, renal failure, or CHD. There was also no notable history of stroke, coronary artery disease, structural cardiac disease, CHD, hypertension, diabetes mellitus, or dyslipidemia in her family.

Palpitation attacks began with exercise or emotional stress, lasting a few hours, and were self‐limited. However, last time, she complained of concomitant shortness of breath. Thereafter, she felt light‐headed and passed out. She was then transferred and admitted to the Emergency room (ER).

### 1.2 Investigations and treatment

**FIGURE 1:**
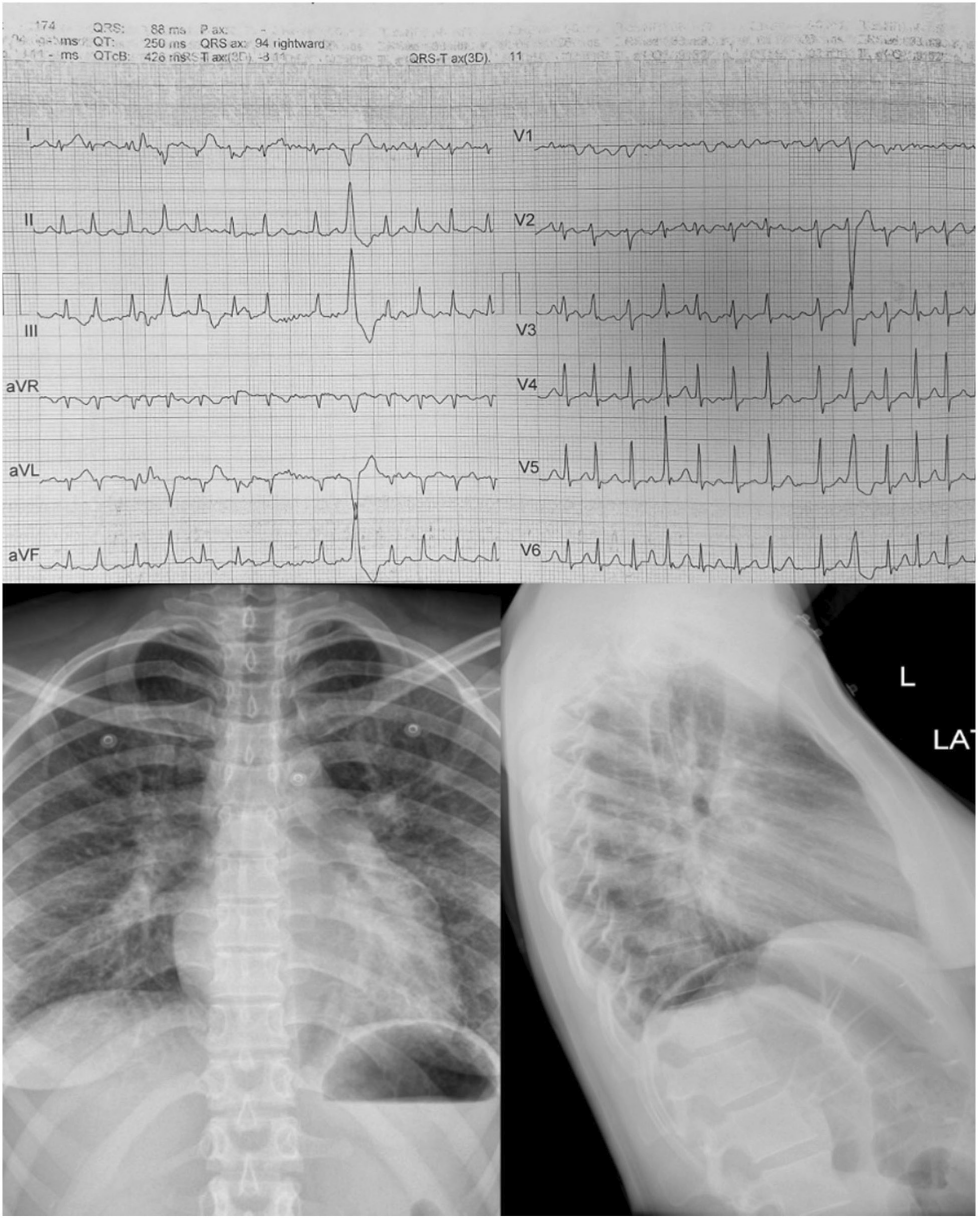
The first electrocardiography (ECG) and chest X‐ray on arrival illustrate atrial fibrillation with a rapid ventricular response on the ECG and normal cardiothoracic ratio with enlargement of the right pulmonary artery and left atrial auricle and hazy opacification consistent with pulmonary venous congestion on the chest X‐ray.

Physical Examination revealed no head injury due to the falling, mild respiratory discomfort, irregular pulse at 180 beats per (BP) minute, and a BP of 133/79 mmHg. The cardiac examination was only remarkable for tachycardic heart sounds, without murmurs. Lung examinations were negative for crackles. Her electrocardiography (ECG; Figure 1) demonstrated atrial fibrillation (AF) with a rapid ventricular response, nonspecific ST‐T changes in inferior leads, and isolated PVC with RVOT origin. Chest X‐ray (Figure 1) showed normal cardiothoracic ratio with enlargement of the right pulmonary artery, left atrial auricle, and hazy opacification consistent with pulmonary venous congestion. A widened carinal angle was also noted with a high‐yield diagnostic clue for obstructive mitral valve pathology.

Transthoracic echocardiography (TTE) revealed a dilated left atrium with left atrial volume index (LAVI) 88 cc/cm^2^ (≤16.5 mL/m^2^ for women), divided with thin fibromuscular membrane into dorsal and ventral chambers, communicate with each other with small size fenestration measured 3 cm in diameter and area of 1.1 cm^2^ in 3D reconstruction with a mean trans membranous pressure gradient of 6 mmHg.

**FIGURE 2:**
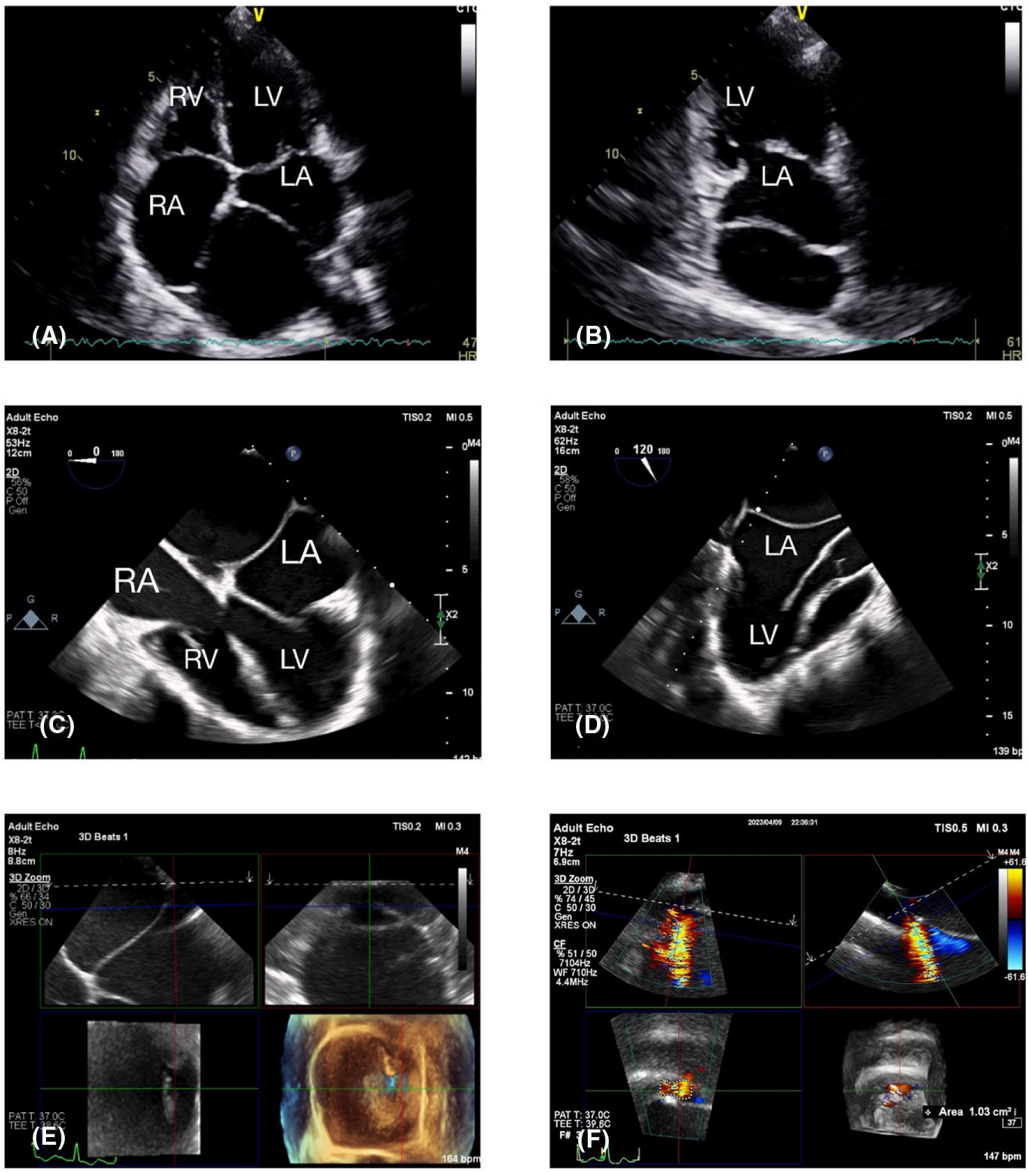
(A–F): Transthoracic and transesophageal echocardiography demonstrated an enlarged left atrium divided with a thin fibromuscular membrane into dorsal and ventral chambers (A–D), which communicate with each other with a small‐size fenestration measured 3 cm in diameter and having an area of 1.1 cm^2^ in 3D reconstruction (E, F).

For further evaluation of the left atrial appendage (LAA) and the presence of concurrent anomalies, the patient also underwent Transesophageal Echocardiography. There were no clots or spontaneous contrast within the left atrial cavity. A dilated mitral valve annulus with an A–P diameter of 46 mm (A–P diameter <35 mm), accompanied by moderate to severe mitral valve regurgitation with a vena contracta of 0.4 cm, was noted. AML was mildly thickened without calcification or prolapse. The probable mechanism of MR seems to be annular dilatation due to congenital Cor triatriatum. Tricuspid and pulmonic valve regurgitation were also noted with a tricuspid regurgitation pressure gradient of 24 mmHg and estimated systolic pulmonary arterial pressure of 30 mmHg (with a normal range of 15–30 mmHg). Biventricular systolic and diastolic functions were preserved. The fenestrated membrane in the left atrium, which divides the cavity into two chambers, was compatible with Cor triatriatum sinistrum type 2 (Figure 2A–F).

To address the patient's AF, we started Oral anticoagulant therapy (OAC) with apixaban 5 mg twice daily, and Rate control was conducted with bisoprolol 5 mg twice daily. The Rhythm control strategy was postponed due to the possible resolution of arrhythmia following the resection of the aberrant membrane. Our patient was then planned for the surgical resection of the membrane and mitral valve repair as the final treatment.

### 1.3 Outcome and follow‐up

Our patient underwent the surgery with no complications. However, in the evaluations following the surgery, the AF rhythm remained. The patient was discharged with OAC after recovery from the surgery. During the next week's follow‐up in the clinic, TTE did not show abnormality other than LA enlargement; however, ECG showed AF rhythm. It was presumed that preserved AF rhythm could be due to the atrial adverse remodeling caused by the CTS. Thus, it was recommended that she continue the OAC. However, 1 month later, she was evaluated again in the clinic, and surprisingly, a sinus heart rhythm was acquired. We decided to perform a 3‐month Holter monitoring while continuing the OAC therapy. Holter monitoring did not show any evidence of AF during these 3 months. Thus, as the mechanical obstruction was eliminated and the AF rhythm was dissolved, OAC therapy was discontinued.

Indication of anticoagulants in patients with this congenital abnormality is a complex issue, and in the context of similar cases, cardiologists may encounter a controversial state for anticoagulation therapy. Consider CTS Per se as an additional risk stratification marker beyond CHA2DS2‐VASc due to the present evidence discussed here.

## 2 DISCUSSION

CTS is considered one of the most uncommon heart abnormalities. The condition is defined by a fibromuscular band that divides the left atrium into two compartments. It has been shown that there is a significant correlation between CTS and increased incidences of AF and stroke. Abnormal anatomy and remodeling of the left atrium may potentially contribute to this elevated risk of clot formation, hence increasing the occurrence of thromboembolic events. It has also been suggested that the process of clot formation in CTS has a comparable etiology to mitral stenosis.^7^ While cardiopulmonary bypass is widely regarded as the preferred surgical treatment, it is important to consider addressing AF in patients.

The primary objectives of clinical therapy for AF encompass the prevention of thromboembolism by the administration of anticoagulant drugs, as well as the preservation of an acceptable heart rate or sinus rhythm through the use of medications or interventional techniques.^8^ OAC is the fundamental approach to preventing thromboembolism.^9^ It is recommended for individuals with AF in the general population, according to the CHA2DS2‐VASc score.^10^ CHA2DS2‐VASc consists of Several clinical risk factors, including age, sex, congestive heart failure, hypertension, diabetes mellitus, stroke/transient ischemic attack, and vascular disease. Furthermore, previous studies have provided evidence indicating that left atrial thrombus is associated with many other variables, including left atrial diameter above 37.5 mm, LAA structure, left ventricular mass, estimated GFR below 60 mL/min/1.73 m^2^, and left ventricular EF.^11^, ^12^

However, the indications for oral OAC in adults with congenital heart disease (ACHD) and AF are not well‐defined, as there are varying guidelines regarding thromboembolic prophylaxis in AF.

Our patient represented AF with palpitation and syncope, but there was neither thrombosis nor spontaneous echo contrast (SEC) in echocardiography, and the CHA2DS2‐VASc score was 1 (woman gender). Regarding stroke risk, even in normal sinus rhythm, there are no currently established guidelines for the anticoagulation management of CTS, which is a challenging approach in this case.

The guidelines for treating AF in patients with ACHD differ notably from the general AF guidelines as well as between themselves. These guidelines are based on a limited level of evidence.^8^, ^13^ This diversity in recommendations can result in a similar diversity in their implementation. Thus, the prescription of OAC for a single lesion may vary significantly depending on the treating physician, leading to changes in the risk of thromboembolic and bleeding events in ACHD patients with AF.

The 2020 ESC Guidelines for the diagnosis and management of AF recommend the consideration of oral anticoagulation in adult patients who have intracardiac repair, cyanosis, Fontan palliation, or systemic right ventricle, and a history of atrial fibrillation, or intra‐atrial re‐entrant tachycardia. Furthermore, it is important to take into account the use of anticoagulation in individuals diagnosed with AF and other congenital heart conditions, particularly when there are one or more non‐sex stroke risk factors.^8^ However, according to the 2023 ACC/AHA/ACCP/HRS Guideline for the Diagnosis and Management of Atrial Fibrillation, it is recommended to administer anticoagulation treatment to adults with AF and moderate or severe forms of CHD. This is especially important for those with low‐flow conditions like Fontan circulation, blind‐ending cardiac chambers, and cyanosis. The OAC therapy should aim to reduce the risk of thromboembolic events in these patients, regardless of the conventional risk score. Nevertheless, CTS has not been clearly classified as mild, moderate, or complex according to this guideline.^13^ Importantly, there have been reports of co‐occurrence of cardioembolic stroke in individuals with cor triatriatum without concurrent AF.^14^

On the other hand, based on previous investigations, it was believed that starting anticoagulants in patients with a low risk of thromboembolic events may only increase the risk of hemorrhagic complications without adding any benefits.^15^ However, recent studies indicate that OAC therapy, even in low‐ and intermediate‐risk populations, is associated with a positive net clinical benefit.^16^, ^17^

Facing the scenario of a CHA2DS2‐VASc score of 1 in a patient with non‐valvular AF is common, and the current opinion statement is the consideration of additional risk factors. The patient's renal function seems to be an important factor in decision‐making; however, our patient's GFR was 75 mL/min, and there was no evidence of chronic kidney disease such as proteinuria.

Echocardiography also helps to identify the risk of stroke. The LAA emptying velocity (<20 cm/s) or fibrosis in the left atrial wall seem to add discriminatory value. An increased left atrial size is also an easily assessable echocardiographic marker that provides the discriminatory value of the patient's additional risk for thromboembolism.^18^ Our patient's left atrial dimension was 48 mm, and LAVI was 88 mL, which was in favor of starting anticoagulation. However, we decided to review the literature on embolic events in adults with Cor triatriatum^7^, ^19^, ^20^, ^21^, ^22^, ^23^, ^24^, ^25^, ^26^, ^27^, ^28^, ^29^, ^30^, ^31^, ^32^ (Table 1).

**TABLE 1:** Cor triatriatum and stroke, reported cases.

Case	Author	Age	Sex	Baseline ECG	Risk factors	Echocardiography	Embolization	Treatment
1	Zaw	48	F	Normal sinus rhythm (NSR)	Hyprttension (HTN), Diabetes Mellitus (DM), Obesity	Obliterated left ventricular cavity with mild mitral regurgitation but normal LV function and a negative bubble study	Right corona radiata	Medical prescription
2	Szabo	18	F	NSR	Unremarkable	Sinister CT, PFO with minimal spontaneous left‐to‐right shunt	Territory of the right posterior cerebral artery	Medical therapy, Dabigatran
3	Diestro	19	F	Unremarkable except for the finding of poor R wave progression	Unremarkable	Mitral valve sclerosis with mild mitral regurgitation, tricuspid valve sclerosis with mild tricuspid regurgitation. Hyperechoic membrane spanning the width of a normal‐sized left atrium (LA), dividing the LA into two communicating chambers. These findings were suggestive of CTS	Right middle cerebral artery (MCA) territory	Medical therapy on ASA
4	Park	55	F	NSR	Unremarkable	A membrane‐like, echo‐dense structure attached to the interatrial septum medially and to the left atrial appendage laterally, which divided the LA into posterior‐superior and anterior‐inferior chambers suggesting a cor triatriatum. There were no apparent pressure gradients within the LA observed using color‐flow doppler imaging	Acute ischemic infarction of the right occipital lobe	Anticoagulation therapy
5	Amara	35	M	NSR	Unremarkable	Transoesophageal echocardiography (TOE) showed a membrane bisecting the left atrium into superior and inferior chambers. The superior chamber received venous return from all four pulmonary veins, and the inferior chamber contained the left atrial appendage, confirming CTS. An agitated saline study confirmed a small right‐to‐left intracardiac shunt, though an ASD or PFO was not clearly appreciated	Left M1 and A1 occlusions in a pattern consistent with embolic stroke	Intravenous tissue plasminogen activator. Subsequently, he was taken for mechanical thrombectomy, with successful recanalization, apixaban 5 mg twice
6	Ridjab	44	F	NSR	Unremarkable	CTS	Defects in the posterior parietal territory on both sides and a small defect in the right posterior inferior cerebellar artery territory	Anticoagulation and statin
7	Baris	55	M	No AF	–	CTS	–	Surgical resection of the fenestrated membrane
8	León	NEONATE	Not Available	Not available	Not available	CTD	Not available	Not available
9	Minocha	17	M	AF	Unremarkable	CTS, soft clot was seen in LAA	The right middle cerebral artery territory of the brain	Anticoagulation
10	Siniorakis	64	F	NSR in history and AF at the time of stroke	Unremarkable	Spontaneous ECHO contrast was visualized in both LA chambers. These observations were further validated by transesophageal ECHO, confirming the diagnosis of CTRS with a multiply fenestrated intraatrial membrane	Acute ischaemic infarction of the right occipital lobe in the posterior cerebral artery territory	AF was electrically cardioverted into sinus rhythm and anticoagulation
11	Spengos	55	M	AF	HTN, Hyperlipidemia (HLP), AF on warfarin	CTS, dilatation of the left atrium (55 mm), and slight concentric hypertrophy of the left ventricle, a 3.5–3 1.3‐cm large thrombus was attached to the basis of the anterior cusp of the mitral valve and the free wall of the left atrium	Acute ischemic lesion in the territory of the left middle cerebral artery	Anticoagulation, cardio‐surgical intervention with total membrane excision
12	von Bartheld	62	F	NSR		CTS	–	Transcatheter balloon dilatation of CTS
13	Nishimoto	57	F			CTS	Multiple cerebral infarctions	Medical therapy
14	Takiya	30	M	AF		CT, Left atrial enlargement (LAE), LA thrombus(+;30 × 10 mm)	Right coronary artery (RCA), Right renal artery, popliteal artery	Risk management (RM)
15	Krasemann	39	F			CT, ASD type II	Left‐sided stroke	RM and ASD repair
16	Huang	65	F	AF		LAE, mild to moderate mitral regurgitation (MR), spontaneous echo contrast in accessory LA	Brain swelling, aortic saddle emboli	Emergency thrombectomy

In our literature review, no prospective study with a control group evaluated the outcome of OAC in CTS patients with low CHA2DS2‐VASc scores. The current body of literature contains a limited number of 18 case reports documenting the association between CTS and cerebrovascular events of cardioembolic origin, in addition to the presence of blood stasis or thrombus. Thus, due to the limited number and heterogenicity of cases, statistical analysis was not feasible.

In five out of 18 cases, SEC or fresh thrombosis was found, all in AF rhythm. Eight out of 18 cases were found in NSR without clot formation. Three cases had concomitant mitral valve regurgitation, and another three cases had simultaneous left to right shunt through Atrial septal defect (ASD)/Patent foramen ovale (PFO). Regarding the reports, AF is not merely the risk factor for embolism in Cor triatriatum, but the membrane bisecting the LA is also responsible.

We only found one case of a 68‐year‐old male with a CHA2DS2VASc score of 1 who was started on OAC.^1^ However, a CHA2DS2VASc score of 1 is an indication of OAC in male patients according to current ESC guidelines, while in our female patient, a score of 1 was due to gender, which is not an indication of OAC by itself. In another case, an 18‐year‐old woman with the presentation of ischemic stroke was found to have CTS.^33^ The CHA2DS2‐VASc score was not calculated in this case; however, due to the Female gender and presentation of Cerebrovascular accident (CVA), the score was supposed to be at least two or more. This patient underwent OAT with 150 mg of dabigatran twice daily and was referred for surgery. However, the patient refused the surgery and decided to pursue conservative care, including medical treatment. There was no recurrence of the stroke during her follow‐up period. On the other hand, in another case, a 19‐year‐old woman presented with CTS and recurrent ischemic stroke despite antithrombotic therapy with aspirin and OAC therapy with 5 mg of apixaban twice daily.^21^

This observed variability in outcomes and therapeutic approaches underscores the significance of appropriate patient selection in the context of anticoagulation.

In our clinical scenario, we encounter a controversial concept, starting an anticoagulant with CHA2DS2‐VASc Score 1 in a female patient. Facing this dilemma in special cases of CTS should be individualized and need more investigation.

We concluded that since the cerebrovascular attack incidence in patients with AF and CTS/CTD is close to 6.5%, which in terms of CHA2DS2VASc, is equivalent to a score of 4–5 points, Starting the OAC regardless of the CHA2DS2‐VASc is a logical decision. Moreover, considering other risk factors and the positive clinical outcome of OAC, even in patients with low and moderate risk of thrombosis, also strengthens the rationale of starting OAC in our CTS patient beyond the CHA2DS2‐VASc score.

However, it should be considered that the sole anticoagulation may not be as effective at preventing embolism in CTS as in other conditions, and there are reports of recurrent thromboembolic stroke even after medical therapy with apixaban and enoxaparin. Thus, surgical excision of the fibromuscular diaphragm is the standard of care in most cases of CTS, especially when associated with complex CHD. Continuation of anticoagulants after the operation is another issue that needs further investigation.

## AUTHOR CONTRIBUTIONS

**Alireza Arzhangzade:** Conceptualization; writing – original draft. **Mahmood Zamirian:** Writing – review and editing. **Salma Nozhat:** Conceptualization; writing – review and editing. **Sasan Shafei:** Writing – review and editing. **Roozbeh Narimani Javid:** Writing – review and editing. **Sarvenaz Salahi:** Writing – review and editing. **Soorena Khorshidi:** Project administration; writing – original draft.

## CONFLICT OF INTEREST STATEMENT

The authors declare that they have no known competing financial interests or personal relationships that could have appeared to influence the work reported in this paper.

## ETHICS STATEMENT

Our institution does not require ethical approval to report individual cases.

## CONSENT

Written informed consent was obtained from the patient to publish this report in accordance with the journal's patient consent policy. A copy of the consent form is available for review by the editor of this journal.

## Data Availability

The data that support the findings of this study are available on request from the corresponding author, [S.Kh]. The data are not publicly available due to containing information that could compromise the privacy of research participant.
